# The Identification and Characteristics of miRNAs Related to Cashmere Fiber Traits in Skin Tissue of Cashmere Goats

**DOI:** 10.3390/genes14020473

**Published:** 2023-02-12

**Authors:** Lirong Qiao, Yuanhua Gu, Shiwei Guo, Shiqiang Li, Jiqing Wang, Zhiyun Hao, Yuzhu Luo, Xiu Liu, Shaobin Li, Fangfang Zhao, Mingna Li

**Affiliations:** Gansu Key Laboratory of Herbivorous Animal Biotechnology, College of Animal Science and Technology, Gansu Agricultural University, Lanzhou 730070, China

**Keywords:** miRNAs, cashmere fiber properties, small RNA sequencing, cashmere goats

## Abstract

microRNAs (miRNAs) are involved in the regulation of biological phenomena by down-regulating the expression of mRNAs. In this study, Liaoning cashmere (LC) goats (*n* = 6) and Ziwuling black (ZB) goats (*n* = 6) with different cashmere fiber production performances were selected. We supposed that miRNAs are responsible for the cashmere fiber trait differences. To test the hypothesis, the expression profiles of miRNAs from the skin tissue of the two caprine breeds were compared using small RNA sequencing (RNA-seq). A total of 1293 miRNAs were expressed in the caprine skin samples, including 399 known caprine miRNAs, 691 known species-conserved miRNAs, and 203 novel miRNAs. Compared with ZB goats, 112 up-regulated miRNAs, and 32 down-regulated miRNAs were found in LC goats. The target genes of the differentially expressed miRNAs were remarkably concentrated on some terms and pathways associated with cashmere fiber performance, including binding, cell, cellular protein modification process, and Wnt, Notch, and MAPK signaling pathways. The miRNA-mRNA interaction network found that 14 miRNAs selected may contribute to cashmere fiber traits regulation by targeting functional genes associated with hair follicle activities. The results have reinforced others leading to a solid foundation for further investigation of the influences of individual miRNAs on cashmere fiber traits in cashmere goats.

## 1. Introduction

Cashmere fiber and wool fiber are derived from the secondary and primary hair follicles in the skin tissue of cashmere goats, respectively [1]. Cashmere fiber is famous for its characteristics of delicacy, softness, and elegance [2], and therefore used to make various textiles, such as scarves, shawls, and some fashionable luxury goods. Compared with mohair, wool, and yak hair, cashmere fiber has a lower fiber diameter, resulting in a higher price. It is, therefore, of great significance to ameliorate cashmere fiber yield and properties in cashmere goats. In this context, the identification of molecular pathways that control cashmere fiber traits is of economic importance as it can offer an opportunity to improve cashmere traits.

It has been found that cashmere fiber traits are regulated by a large number of structural genes and non-coding RNAs. These RNAs participate in signaling pathways [3] and then regulate the onset and cessation of hair follicle growth, eventually resulting in changes in cashmere fiber traits. The microRNAs (miRNAs) belong to non-coding RNAs and range in length from 20 to 24 nt [4]. The miRNAs can either accelerate degradation or repress translation of the target mRNAs by specifically binding to their target sites [4]. It was found that miRNAs participate in the regulation of many biological phenomena, including ontogenesis, organ growth, cell activities, hair follicle development, and pigmentation [5,6].

The miRNAs are widely expressed in the skin of a range of mammals, such as mice, goats, and sheep [7,8,9], suggesting that miRNAs may be responsible for hair growth and development in mammals. Some studies have further confirmed the important roles of individual miRNA in the regulation of hair fiber traits. For example, miR-31 contributed to the morphogenesis of hair follicles and shafts in mouse skin [10]. The miR-22 can promote apoptosis of hair follicles and repress keratinocyte differentiation in mice skin [11]. The miR-214 inhibited inducible keratinocyte proliferation, eventually resulting in fewer hair follicles with decreased hair bulb size in mice [12]. The roles of miR-205 [13] and miR-125b [7] in the activities of hair follicle stem cells were also reported.

Up to now, there are some literatures that have reported miRNA expression profiles of skin tissue in goats, but they were mainly carried out using skin samples collected from different development stages or compared differences of miRNAs between goats with other species. For example, Liu et al. [14] found 44 differentially expressed miRNAs during anagen when compared to telogen in Inner Mongolian cashmere goats. Their target genes were concentrated on Jak-STAT, Notch, and Wnt signaling pathways that were correlated with hair follicle morphogenesis. Li et al. [15] compared the skin miRNA profiles of LC goats with a fine-wool sheep breed during the telogen phase of the fiber and found that the target mRNAs of differentially expressed miRNAs were concentrated on Notch, TGF-β, Wnt, and MAPK signaling pathways. However, there is few literatures comparing the miRNA expression in skin tissues between different goat breeds.

Liaoning cashmere (LC) goat is an important cashmere goat breed in China outstanding for its high cashmere yield. The average cashmere fiber yield of adult bucks and does is 1300 g and 640 g, respectively [16]. Meanwhile, they had an average fiber diameter of 16.7 μm and 15.5 μm, respectively. Compared to LC goats, Ziwuling black (ZB) goats distributed in Qingyang City (Gansu Province, China) have lower cashmere quantity and better quality of cashmere fiber. Briefly, the yield and diameter of cashmere fiber of ZB goats are 310 g and 14.1 μm, respectively, on average. Despite there being a significant difference in cashmere fiber traits between LC goats and ZB goats, the biological mechanism underlying these differences is imprecision. In this study, small RNA-sequencing (RNA-seq) was performed to compare differences in the skin miRNA expression profiles between LC goats and ZB goats. The differentially expressed miRNAs between the caprine goat breeds and functional pathways related to cashmere fiber traits were also identified. The results will establish a solid underpinning for realizing the influences of miRNAs in caprine cashmere fiber quantity and quality.

## 2. Materials and Methods

### 2.1. Goats Investigated and Samples Collection

Twelve healthy three-year-old male goats were chosen from Yusheng Cashmere Goat Breeding Company in Huan County, Gansu Province, China, including six LC goats and six ZB goats. These goats were raised under the same nutrition levels and management conditions. The cashmere yield of the LC goats and ZB goats were 1539 ± 26.6 g and 395 ± 17.5 g, respectively. The LC goats also have a higher cashmere fiber diameter (15.89 ± 0.07 μm) than ZB goats (13.9 ± 0.03 μm). Meanwhile, the LC goats and ZB goats produce white and dark purple cashmere fiber, respectively.

During the growth phase of the cashmere fiber, the skin samples for the twelve goats were gathered using surgical biopsy. Briefly, cashmere and wool fibers that grew at the lateral part of the right mid-side of the body were removed, and the parts were sterilized twice with alcohol. After local anesthesia, skin samples with a size of 3 cm × 2 cm were collected. The specimens were washed with normal saline and RNase water. Finally, the samples were cut into a piece of 1 cm × 2 cm and then quickly placed in liquid nitrogen.

Total RNA from 12 caprine skin tissue samples was extracted using a Trizol reagent kit (Invitrogen, Carlsbad, CA, USA). The quantity and quality of the RNA were examined using a NanoDrop 8000 spectrophotometer (NanoDrop Technologies, Wilmington, DE, USA). The RNA integrity number (RIN) was also assessed using Agilent 2100 Bioanalyzer (Agilent Technologies, Santa Clara, CA, USA). The 12 RNA samples with a RIN > 7 were prepared for small RNA-seq.

### 2.2. Small RNA Sequencing

The RNA with a range of 18–30 nt in size was concentrated by polyacrylamide gel electrophoresis. Subsequently, the 3′ adapters were inserted into RNA, and enrichment was then performed for the RNA with 36–44 nt in length. The 5′ adapters were then ligated to the RNAs as well. The products were reverse transcribed by reverse transcription-polymerase chain reaction (RT-PCR), and the PCR products with 140–160 bp in size were used to generate cDNA libraries. Agilent 2100 and RT-quantitative PCR (RT-qPCR) were carried out to assess the libraries’ quality. The small RNA-seq was finally carried out using an Illumina HiSeqTM4000 sequencer (Illumina, San Diego, CA, USA).

### 2.3. Processing of Small RNA-Seq Data

To get clean reads, the low-quality reads were removed from the raw reads using fastp v0.18.0, including the reads without 3′ adapters, the reads containing polyA (more than 70% of the base of a read was A), the reads without inserted fragments, and reads with <18 nt. Subsequently, the reads processed were aligned to the GenBank database v209.0 and Rfam v11.0 databases to take out other RNA from the samples, including ribosomal RNA (rRNA), small cellular RNA (scRNA), small nucleolar RNA (snoRNA), small nuclear RNA (snRNA), and transfer RNA (tRNA). The reads obtained were then aligned to goat Genome ARS1.2 (https://www.ncbi.nlm.nih.gov/assembly/GCF_001704415.2, accessed on 29 May 2020) to delete exons, introns, and repeat sequences. The remaining reads were mapped to miRBase v22.0 database to obtain known miRNAs in goats (named known caprine miRNAs) and other species (named known species-conserved miRNAs). Finally, miReap v.0.2 [17] was used to obtain the possible new miRNAs (named novel miRNA) by predicting their special secondary structure. To make the annotation unique for each small RNA, the order of priority was as follows: rRNA >known caprine miRNA > known miRNA edit > known species-conserved miRNA > repeat sequence > exon sequence > novel miRNA > intron sequence.

The abundance of miRNAs was standardized using transcripts per million (TPM). Differential expression analysis of miRNAs was performed in skin samples between the two caprine breeds using DESeq v2.0 [18]. Only miRNAs with |Fold Change| > 2.0 and *p*-value < 0.05 was defined as differentially expressed.

### 2.4. Role Study of Differentially Expressed miRNAs

Miranda v3.3a [19] and TargetScan v7.0 [20] were carried out to calculate the target genes of differentially expressed miRNAs, and the results originated from the software were then overlapped. The roles of the target genes were annotated using Gene Ontology (GO) and the Kyoto Encyclopedia of Genes and Genomes (KEGG) databases. GO terms or pathways with *p* < 0.05 in the hypergeometric test were defined as significantly enriched pathways. A total of 14 miRNAs were chosen to build a miRNA-mRNA figure using Cytoscape v3.5.1 [21].

### 2.5. RT-qPCR

The random selection of 17 differentially expressed miRNAs in skin tissue was applied for RT-qPCR. The RNA samples originally extracted for small RNA-seq analysis were used to produce cDNA using SuperScript II reverse transcriptase (Invitrogen, Carlsbad, CA, USA). The U6 and 18sRNA were selected as internal reference genes [6,22]. The abundance of the miRNAs was quantified in triplicate using 2 × ChamQ SYBR qPCR (Nanjing Vazyme, China) and then calculated using a 2^−ΔΔCt^ method [23]. The sequence information of primers is shown in Supplementary File S1.

## 3. Results

### 3.1. Small RNA-Seq Data

A total of 13,820,430 and 14,638,317 raw reads were generated in the skin tissue samples of LC goats and ZB goats, respectively. The raw reads produced in this investigation have been submitted to the GenBank database with accession numbers SRR19505486–SRR19505497. After filtering out the low-quality reads, 13,552,198 and 14,398,090 clean reads were attained from the two breeds on average. Of the small RNA reads obtained in the study, the vast majority of reads ranged from 20 to 24 nt in length. The miRNAs with 22 nt in length were the most common, with a proportion of 37.0% and 40.0% in LC goats and ZB goats, respectively. This was followed by miRNAs with 21 nt in length, accounting for 25.5% and 28.1%, respectively (Figure S1).

### 3.2. Characteristics of miRNAs

**Figure 1:**
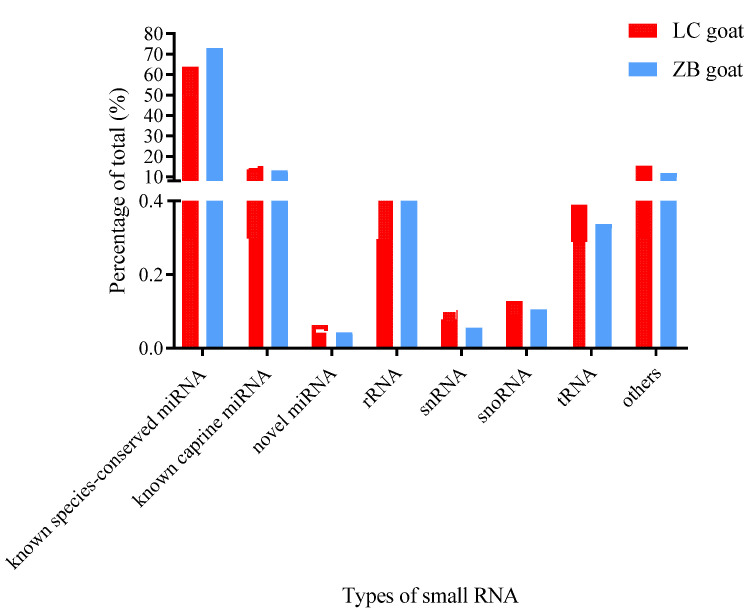
The proportion of small RNAs in the skin tissue samples of Liaoning cashmere (LC) goats and Ziwuling black (ZB) goats.

A total of 1293 miRNAs were expressed in the caprine skin samples, including 399 known caprine miRNAs, 691 known species-conserved miRNAs, and 203 novel miRNAs (Supplementary File S2). Among them, 1278 miRNAs were co-expressed in both the two goat breeds, while five and ten miRNAs were only expressed in LC goats and ZB goats, respectively. Of the small RNAs of the two breeds annotated, the known species-conserved miRNAs were the most abundant, with a proportion of 63.7% and 72.9% in LC goats and ZB goats, respectively (Figure 1).

Among all the miRNAs identified, miR-26a-5p has the highest expression with a TMP value of 112,864 and 136,978 in LC goats and ZB goats, respectively, followed by miR-27b-3p, miR-199a-5p, miR-143-3p, and miR-99a-5p (Table 1).

### 3.3. Screening of Differentially Expressed miRNAs and Verification of Sequencing Results

**Figure 2:**
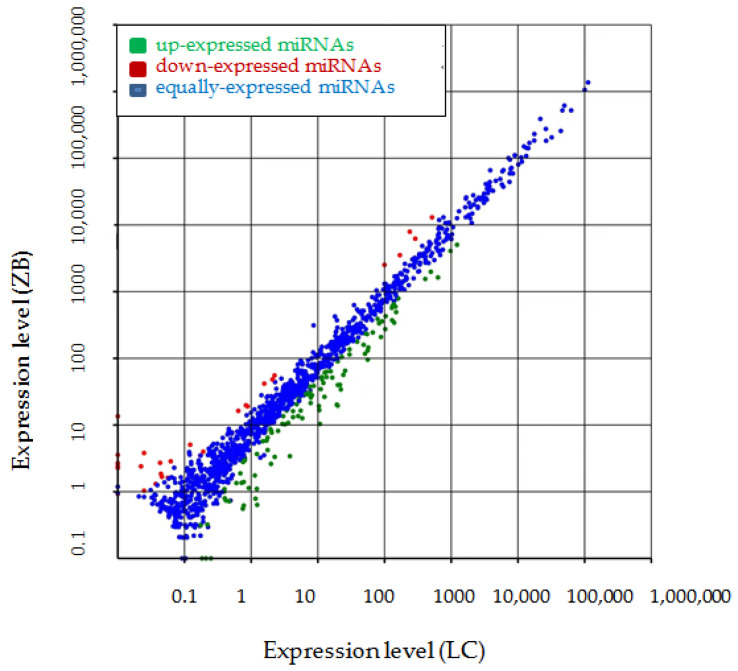
Scatter plot of expressed miRNAs in two cashmere goat breeds.

A total of 144 differentially expressed miRNAs were found in skin tissue samples, including 112 up-regulated miRNAs and 32 down-regulated miRNAs in LC goats compared to ZB goats (Figure 2, Supplementary File S3). Of the miRNAs, miR-2137 was the most up-regulated, followed by miR-1260, miR-9277, miR-6596-5p, and miR-2478. The miR-486-5p was the most down-regulated, followed by miR-124-3p, miR-451-5p, miR-486, and miR-144-3p (Supplementary File S3).

**Figure 3:**
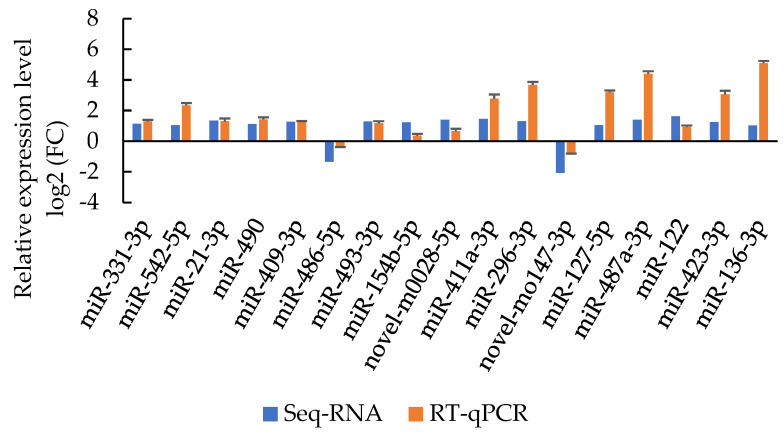
The comparison of results from RT-qPCR with those from small RNA-seq in skin tissue samples for 17 miRNAs. The error bars indicate the standard deviation of the mean values of three technological replicates for each sample.

In order to test the small RNA-seq results, the abundance of 17 differentially expressed miRNAs screened from small RNA-seq were further detected using RT-qPCR analysis. After comparison, the RT-qPCR results were identical to those originated from small RNA-seq (Figure 3). These results further confirm the reliability and repeatability of small RNA-seq results.

### 3.4. Role Study of miRNAs Screened

**Figure 4:**
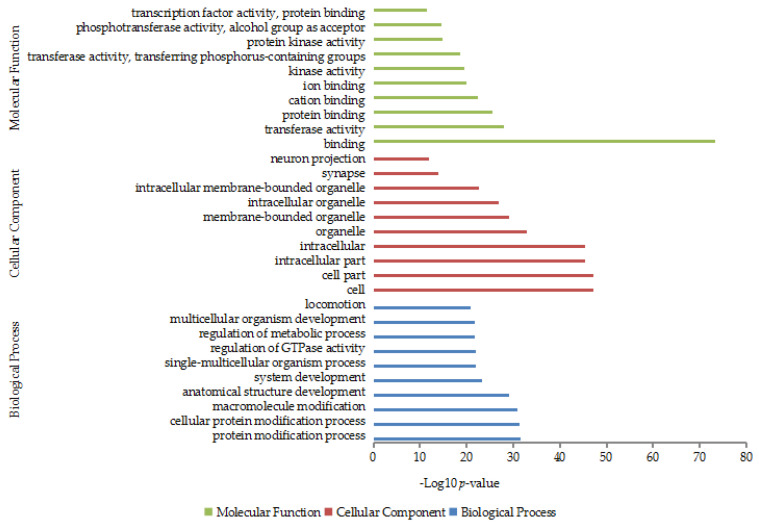
The top 10 terms in each Gene Ontology (GO) category concentrated by the target genes. The X-axis and Y-axis show the calculated results of -Log10 (*p*-value) and title of GO terms, respectively.

The target mRNAs of the differentially expressed miRNAs were remarkably concentrated on 1237 GO terms (Supplementary File S4). Of the terms, binding (*p* = 4.74 × 10^−74^) was the most enriched term, followed by cell (*p* = 7.27 × 10^−48^), cell part (*p* = 7.61 × 10^−48^), intracellular part (*p* = 3.61 × 10^−46^), intracellular (*p* = 3.83 × 10^−46^) and organelle *(p* = 1.31 × 10^−33^) (Figure 4).

**Figure 5:**
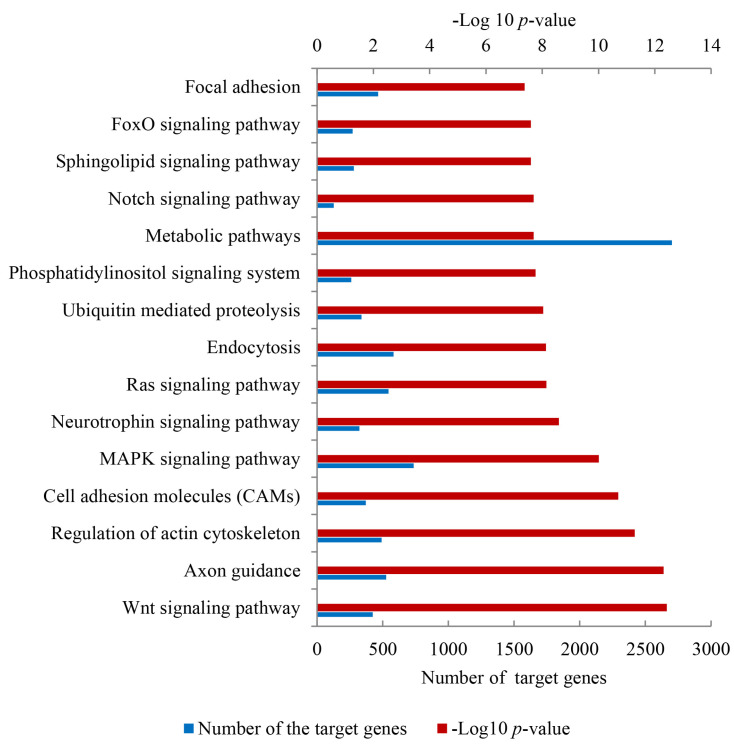
The top 15 KEGG signaling pathways in the skin samples. The upper X-axis and X-axis below represents the calculated results of −Log10 (*p*-value) and the amount of the target mRNAs, respectively.

To further explain functional pathways in which these target genes may be involved, a KEGG pathway enrichment analysis was carried out. The target genes were significantly concentrated on 166 KEGG pathways (Supplementary File S4). Of these pathways, the Wnt signaling pathway was the most significant pathway (*p* = 1.07 × 10^−15^), followed by Axon guidance (*p* = 2.77 × 10^−15^), regulation of actin cytoskeleton (*p* = 5.86 × 10^−14^), cell adhesion molecules (CAMs) (*p* = 2.83 × 10^−13^) and MAPK signaling pathway (*p* = 1.66 × 10^−12^). It was notable that some pathways related to hair follicle development were also enriched, including Wnt, MAPK, and Notch signaling pathways. The most significant 15 KEGG pathways are exhibited in Figure 5.

### 3.5. The miRNA Binding Sites Analysis of mRNAs

**Figure 6:**
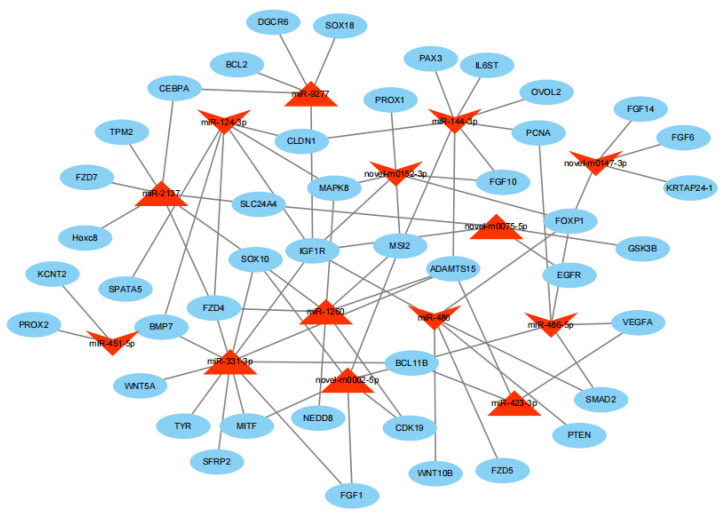
The miRNA-mRNA interaction figure. The red triangles and inverted triangles indicate up-regulated and down-regulated miRNAs in the skin tissue samples of LC goats when compared to ZB goats, respectively. Blue circles represent the target genes.

Upon the joint prediction of Miranda v3.3a and TargetScan v7.0, a total of 15,486 potential target mRNAs were obtained for the 144 differentially expressed miRNAs. To clearly demonstrate the sponge effect of miRNA on mRNA, seven up-regulated miRNAs and seven down-regulated miRNAs in LC goats were further selected compared to ZB goats, including the three most up-regulated known species-conserved miRNAs (miR-2137, miR-1260, and miR-9277), two most up-regulated known caprine miRNAs (miR-423-3p and miR-331-3p), two most up-regulated novel miRNAs (novel-m0075-5p and novel-m0002-5p), three most down-regulated known species-conserved miRNAs (miR-486-5p, miR-124-3p, and miR-486), two most down-regulated known caprine miRNAs (miR-451-5p and miR-144-3p), and two most down-regulated novel miRNAs (novel-m0147-3p and novel-m0152-3p). There were 9,190 target mRNAs predicted for the 14 miRNAs. Among the target mRNAs predicted, some genes associated with cashmere fiber color and hair follicle morphogenesis were selected to construct an miRNA-mRNA interaction network, including keratin association protein gene 24-1 (*KRATP24-1*), melanocyte inducing transcription factor (*MITF*), tyrosinase (*TYR*), sulfite oxidase factor 10 (*sox10*), sulfite oxidase factor 18 (*sox18*), ovo like zinc finger 2 (*OVOL2*), insulin like growth factor 1 receptor (*IGF1R*), phosphatase and tensin homolog (*PTEN*), and proliferating cell nuclear antigen (*PCNA*) (Figure 6).

## 4. Discussion

This study mainly compared caprine skin tissue miRNA expression profiles of LC goats with ZB goats producing less black cashmere fiber with decreased fiber diameter. Our observation that miRNAs with 22 nt were the most abundant in caprine skin was consistent with the findings in sheep and goats [24,25,26,27]. In addition, of all the small RNAs identified, known species-conserved miRNAs accounted for the largest proportion. This result resembles what was described by Liu et al. [8], who identified 22 novel miRNAs and 68 and 248 known miRNAs in goats and other species, respectively.

Among the miRNAs with the highest expression in caprine skin tissue, most of them were associated with hair follicle activities. For example, as the miRNA with the highest expression level in both caprine breeds, miR-26a-5p has been found to accelerate caprine dermal papilla cell proliferation which promotes hair follicle morphogenesis [28]. Kandyba et al. [29] also found that miR-26a promoted hair follicle periodic growth and development and pigmentation via the TGF-β/SMAD pathway. As another highly expression miRNA, the essential roles of miR-199a-5p in hair follicle activities have been reported. The miR-199a can directly target the Wnt signaling pathway to promote hair follicle morphogenesis in goats. The Wnt pathway is responsible for hair follicle development [30,31]. The inhibition of the Wnt pathway destroyed hair follicle structure [32]. Besides miR-26a-5p and miR-199a-5p, miR-143-3p was also involved in hair follicles development cycles. The higher expression of miR-143 was detected the in small wavy villi of Hu sheep with more secondary hair follicles [33]. This indicates that miR-143 can promote cashmere fiber yield by increasing the amount of secondary hair follicles.

It was notable that miR-486-5p was the most down-regulated miRNA in LC goats. Although the role of miR-486-5p in caprine hair follicles development and cashmere fiber traits is unknown, our miRNA-mRNA figure showed that miR-486-5p would bind with multiple genes associated with caprine hair follicles cycle, including vascular endothelial growth factor A (*VEGFA*) and B cell lymphoma/leukemia 11B (*BCL11B*) (Figure 6). *VEGFA* can accelerate cashmere fiber growth by increasing the area of vibrissa follicles, hair follicles, and hair shafts during the murine hair cycle [34]. Meanwhile, the loss of *BCL11B* diminished hair follicle density during the embryonic period in mice [35]. These results may explain why miR-486-5p had a lower abundance in LC goats with higher cashmere fiber yield, as it less inhibited the promoted effect of *VEGFA* and *BCL11B* on growth, size, and density of hair follicles.

Of the down-regulated miRNAs in LC goats identified, miR-129-5p attracted our attention. The regulator effect of miR-129-5p on melanin biosynthesis in mammals has been analyzed. The miRNA influenced coat color generation by decreasing the expression of *TYR* [36]. The miR-129-5p has been confirmed to bind with *TYR* and *MITF*. The two genes can affect the formation of melanin [37]. Wu et al. [38] also found that miR-137 in black goats promoted melanin formation by targeting *MITF* and *TYR*. The results may explain why miR-129-5p had lower expression in LC goats with white cashmere fiber.

Of 203 novel miRNAs found, ten miRNAs were differentially expressed miRNAs. It was noteworthy that novel-m0147-3p would target *KRTAP24-1* (Figure 6). The gene encodes a high-sulphur keratin-associated protein that is one of the main structural ingredients of cashmere fibers. Variations in *KRTAP24-1* were found to correlate with the diameter of cashmere fiber in cashmere goats [39]. It was therefore inferred that novel-m0147-3p might be responsible for the diameter difference of cashmere fiber between LC goats and ZB goats by targeting KRTAP24-1. In addition, novel-m0152-3p would target musashi 2 (*MSI2*) (Figure 6). Studies have shown that *MSI2* can inhibit hair growth and preserve the condition of the rest of hair follicle stem cells [40]. This may be the reason for the difference in cashmere fiber yield between the caprine breeds.

Of the seven up-regulated miRNAs that appeared in the miRNA-mRNA network, only novel-m0001-3p and novel-m0002-5p had higher abundance, while the others were expressed at relatively low levels. The novel-m0002-5p would potentially target *Sox10* and *MITF* (Figure 6). *MITF* can promote the formation of melanin [41]. *Sox10* can promote the development of melanocytes, and the gene can also regulate the transcription of *MITF*, thereby regulating the development of melanocytes [42]. Therefore, the abundance of novel-m0002-5p may contribute to the difference in fiber color between the two breeds.

It was found in GO annotation that the target mRNAs were remarkably concentrated in the terms of binding, cell, and cellular protein modification process. These may participate in cell proliferation, development, and other physiological processes. What is more important, they change the cashmere fibers’ growth cycle by regulating the abundance of related proteins in cells [14]. As might be expected, the Wnt signaling pathway had the lowest *p*-value for the target mRNAs. Besides, MAPK and Notch signaling pathways were also concentrated by the target genes. The Wnt signaling pathway is one of the pivotal pathways for hair follicle onset, development, and regeneration [43]. It mainly promotes the activities of hair follicle stem cells and plays a crucial role in all types of hair follicle morphogenesis [44,45]. Stem cell factors contribute to melanin synthesis. When its ligand binds with the receptor, it stimulates MAPK reaction to phosphorylate MITF, which blocks the synthesis of melanin [46]. This suggests that the MAPK signaling pathway regulates the difference in the color of cashmere fiber between LC goats and ZB goats. The Notch signaling pathway can ensure the normal development of hair stem by promoting cell differentiation and accelerating hair follicles growth and development [47].

## 5. Conclusions

A total of 144 miRNAs were differentially expressed in caprine skin samples with remarkably different cashmere fiber traits. Their target genes were concentrated on important GO terms and KEGG pathways correlated with hair follicle activities. These results may contribute to further investigation into the influence of miRNAs on the yield, diameter, and color of cashmere fiber. They also provide new ideas for regulating the development of cashmere goat follicles and improving the yield and quality of cashmere fiber by manipulating the abundance of the differentially expressed miRNAs identified or their target mRNAs.

## Figures and Tables

**Table 1:** The top ten most expressed miRNAs.

Name	Sequence	TPM-Liaoning Cashmere Goats	TPM-Ziwuling Balck Goats	Type
miR-26a-5p	TTCAAGTAATCCAGGATAGGCT	112,864	136,978	Known caprine miRNA
miR-27b-3p	TTCACAGTGGCTAAGTTCTGC	100,446	106,071	Known caprine miRNA
miR-199a-5p	CCCAGTGTTCAGACTACCTGTTC	62,866	52,205	Known caprine miRNA
miR-143-3p	TGAGATGAAGCACTGTAGCTCG	49,715	61,548	Known caprine miRNA
miR-99a-5p	AACCCGTAGATCCGATCTTGT	46,392	52,219	Known caprine miRNA
miR-10b-5p	TACCCTGTAGAACCGAATTTGT	21,680	38,877	Known caprine miRNA
let-7a-5p	TGAGGTAGTAGGTTGTATAGTT	26,241	27,468	Known caprine miRNA
miR-125b-5p	TCCCTGAGACCCTAACTTGT	32,068	20,536	Known caprine miRNA
miR-24-3p	TGGCTCAGTTCAGCAGGAAC	17,643	23,069	Known caprine miRNA
miR-23a	ATCACATTGCCAGGGATTTCC	17,566	18,377	Known caprine miRNA

## Data Availability

The data presented in this study are available on request from the corresponding author. The raw reads obtained in this study have been deposited in GenBank database with accession numbers SRR19505486-SRR19505497.

## Supplementary Materials

The following supporting information can be downloaded at: https://www.mdpi.com/article/10.3390/genes14020473/s1. Figure S1: Nucleotide length distribution of small RNA fragments in skin tissue samples of Liaoning cashmere (LC) goats and Ziwuling black (ZB) goats. Supplementary File S1: PCR primers used for RT-qPCR. Supplementary File S2: The miRNAs identified from skin tissue samples of Liaoning cashmere (LC) goats and Ziwuling black (ZB) goats. Supplementary File S3: The up-regulated and down-regulated miRNAs identified from skin tissue samples of Liaoning cashmere (LC) goats when compared to Ziwuling black (ZB) goats. Supplementary File S4: The GO and KEGG pathways significantly enriched by the target genes of differentially expressed miRNAs identified from skin tissue samples of Liaoning cashmere (LC) goats and Ziwuling black (ZB) goats.
