# Supplementary material for: The Identification and Characteristics of miRNAs Related to Cashmere Fiber Traits in Skin Tissue of Cashmere Goats
Source: Genes (Basel). 2023 Feb 12;14(2):473. doi: 10.3390/genes14020473 (PMC9957446; doi:10.3390/genes14020473)
Supplement: Supplementary file 1 [file genes-14-00473-s001.zip › Figure S1. Nucleotide length distribution of small RNA fragments in skin tissue samples of Liaoning cashmere (LC) goats and Ziwuling black (ZB) goats.pdf]

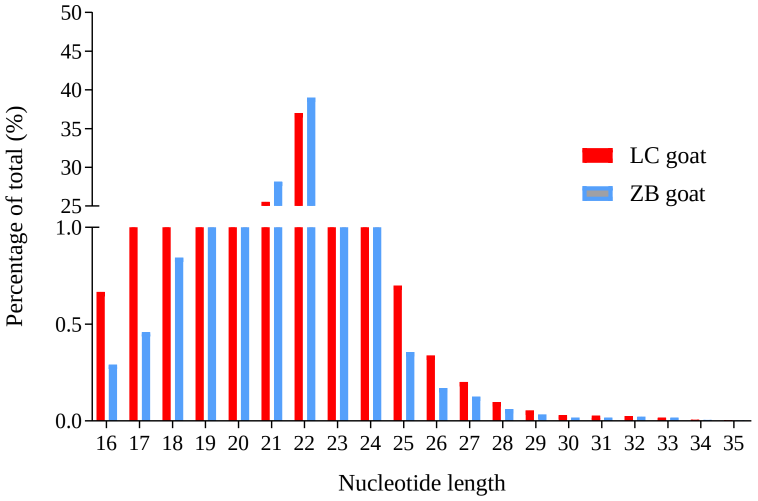

Figure S1. Nucleotide length distribution of small RNA fragments in skin tissue samples of Liaoning cashmere (LC) goats and Ziwuling black (ZB) goats.
