# Supplementary material for: The Identification and Characteristics of miRNAs Related to Cashmere Fiber Traits in Skin Tissue of Cashmere Goats
Source: Genes (Basel). 2023 Feb 12;14(2):473. doi: 10.3390/genes14020473 (PMC9957446; doi:10.3390/genes14020473)
Supplement: Supplementary file 1 [file genes-14-00473-s001.zip › Supplementary File S1. PCR primers used for RT-qPCR.pdf]

Supplementary File S1. PCR primers used for RT-qPCR

RNA	Forward (5'→3')	Reverse (5'→3')
miR-122	TGGAGTGTGACAATGGTGTTTG	mRQ 3′ primer [1]
miR-127-5p	GAAGCTCAGAGGGCTCTGATTC	mRQ 3′ primer
miR-486-5p	TCCTGTACTGAGCTGCCCCGAG	mRQ 3′ primer
miR-136-3p	ATCATCGTCTCAAATGAGTCT	mRQ 3′ primer
miR-154b-5p	AGAGGTCTTCCATGGTGCATTC	mRQ 3′ primer
miR-21-3p	CAACAGCAGTCGATGGGCTGT	mRQ 3′ primer
miR-296-3p	AGGGTTGGGCGGAGGCTTTCCT	mRQ 3′ primer
miR-331-3p	CCCCTGGGCCTATCCTAGAAC	mRQ 3′ primer
miR-411a-3p	TATGTAACACGGTCCACTAAC	mRQ 3′ primer
miR-423-3p	AGCTCGGTCTGAGGCCCCTCAGT	mRQ 3′ primer
miR-409-3p	GAATGTTGCTCGGTGAACCCC	mRQ 3′ primer
miR-487a-3p	AATCATACAGGGACATCCAGTT	mRQ 3′ primer
miR-490	CAACCTGGAGGACTCCATGCTGT	mRQ 3′ primer
miR-493-3p	TGAAGGTCTACTGTGTGCCAGG	mRQ 3′ primer
miR-542-5p	TCGGGGATCATCATGTCACGAGA	mRQ 3′ primer
novel-m0147-3p	AGGCACCTAGGCTGGAGACC	mRQ 3′ primer
novel-m0028-5p	TGTGGACACATGGATGCACATG	mRQ 3′ primer
U6	GGAACGATACAGAGAAGATTAGC	TGGAACGCTTCACGAATTTGCG
18sRNA	GTGGTGTTGAGGAAAGCAGACA	TGATCACACGTTCCACCTCATC
